# Roles of the screw types, proximity and anterior band wiring in the surgical fixation of transverse patellar fractures: a finite element investigation

**DOI:** 10.1186/s12891-019-2474-7

**Published:** 2019-03-04

**Authors:** Chih-Hsien Chen, Yen-Nien Chen, Chun-Ting Li, Chih-Wei Chang, Chih-Han Chang, Yao-Te Peng

**Affiliations:** 1 Department of BioMedical Engineering, National Cheng Kung University, Tainan City, Taiwan; 2 Department of Orthopaedic Surgery, Tainan Municipal Hospital (Managed by Show Chwan Medical Care Corporation), Tainan City, Taiwan; 3 Department of Orthopedics, Show Chwan Memorial Hospital, Changhua City, Taiwan; 4 Graduate Institute of Mechatronic System Engineering, National University of Tainan, Tainan City, Taiwan; 5 Department of Orthopedics, College of Medicine, National Cheng Kung University, Tainan City, Taiwan; 6 Department of Orthopedics, National Cheng Kung University Hospital, College of Medicine, National Cheng Kung University, Tainan City, 701 Taiwan; 7 Metal Industries Research & Development Centre, Kaohsiung City, Taiwan

**Keywords:** Patellar fracture, Screw thread, Screw proximity, Wire, Minimally invasive surgery, Finite element method

## Abstract

**Background:**

Cannulated screws with an anterior wire are currently used for managing transverse patellar fracture. However, the addition of anterior wiring with various types of screws via open surgery to increase the mechanical stability is yet to be determined. Hence, this study aimed to compare the mechanical behaviors of a fractured patella fixed with various screws types and at various screw locations with and without the anterior wire. The present study hypothesized that using the anterior wire reduces the fracture gap formation.

**Methods:**

A finite element (FE) model containing a fractured patella fixed with various types of cannulated screws and anterior wiring was created in this study. Three types of screws, namely partial thread, full thread, and headless compression screws, and two screw depths, namely 5 and 10 mm away from the anterior surface of the patella, were included. The effect of the anterior wire was clarified by comparing the results of surgical fixation with and without the wire. Two magnitudes and two loading directions were used to simulate and examine the mechanical responses of the fractured patella with various fixation conditions during knee flexion/extension.

**Results:**

Compared with partial thread and headless compression screws, the full thread screw increased the stability of the fractured patella by reducing fragment displacement, fracture gap formation, and contact pressure while increasing the contact area at the fracture site. Under 400-N in the direction 45°, the full thread screw with 5-mm placement reduced the gap formation by 86.7% (from 2.71 to 0.36 mm) and 55.6% (from 0. 81 to 0. 36 mm) compared with the partial thread screw with 10-mm placement, respectively without and with the anterior wire.

**Conclusion:**

The anterior wire along with the full thread screw is preferentially recommended for maintaining the surgical fixation of the fractured patella. Without the use of anterior wiring, the full thread screw with 5-mm placement may be considered as a less invasive alternative; however, simple screw fixation at a deeper placement (10 mm) is least recommended for the fixation of transverse patellar fracture.

## Background

Patellar fracture accounts for 1% of all skeleton fractures [1–3], with an incidence of 13.5/100,000 person-years [4]. For displaced patellar fractures, surgical fixation is often required to maintain the reduction of the fractured patella and the function of extension mechanism of the lower extremity [5–7]. In 1950s, the AO group introduced the concept of the modified anterior tension band; improved stability as well as reliable outcomes made which the treatment of choice while treating transverse patellar fractures. However, certain complications like wire migration, loss of reduction and implant irritation are not uncommon [8–13].

Due to the inherent design and enhanced strength, a screw with threading works better than the smooth Kirschner wires as a supportive device while preventing fragment separation [14, 15]. Recently, surgeons preferred the use of cannulated screws along with an anterior wire in a figure of eight for fracture fixation based on a higher mechanical stability obtained by this modification [14, 16, 17]. In this technique, two cannulated screws are used rather than the two conventional Kirschner wires to increase the structural stiffness and subsequently the stability of the fractured patella [18].

On the other hand, to create an effective tension band, the anterior wire in a figure of eight is often applied via an open approach which may increase the surgical trauma and compromise the following biological healing. Although fixation using anterior wire increases the stability of the fractured patella more substantially than that without anterior wire, a less invasive fixation alternative using screws without the anterior wire was thus proposed based on the supporting strength from screws. Without the anterior tension wiring, these minimally invasive surgeries (MIS) were expected to reduce early postoperative pain, results in higher mobility angles of the injured knee, and decreases the incidence of complications [19]. However, to our note, the mechanical stability of fractured patella without the anterior wire was not fully investigated. Options between different screw types as well as their optimal locations were yet to be determined. For example, full thread or headless compression screws, are common devices available for increasing the stability of fracture fixation, however, they were less considered in the literature about the fixation of patellar fractures.

In addition to the mechanical stability, the contact status including the contact area and pressure at the fracture site is a concern critical for surgeons to choose the suitable fixation strategy. However, it is difficult to determine this issue under current laboratory setting due to the difficult embedding minute sensors without disruption the bone and wires. To solve the mentioned problem, the finite element (FE) analysis, a numerical method that does not require sensors, provides a practical method for calculating the contact pressure and area as well as internal stress [20–22]. By using the FE method, this study aimed to investigate the mechanical behaviors, including fracture gap formation, fragment displacement, and contact pressure and area at the fracture site, of a fractured patella fixed with various screw types, proximity and the use of anterior wire or not. In the present study, the use of anterior wire is applied through an open approach and is hypothesized to achieve the best stability among all kinds of screws configuration while reducing the fracture gap. However, the mechanical strength achieved by the screw fixation only is to be considered as a less invasive fixation alternative without anterior wiring.

## Methods

An FE model comprising a fractured patella fixed with various types of cannulated screws with and without the anterior wire was developed based on our previous model [23]. Furthermore, two screw proximities, namely 5 and 10 mm away from the anterior surface of the patella, were considered in the simulation. The screw types used were full thread, partial thread, and headless compression screws. The anterior wire was excludes to represent MIS procedure in the fixation of patellar fracture, while the anterior wire was used to represent open surgery.

### Solid modeling

**Fig. 1:**
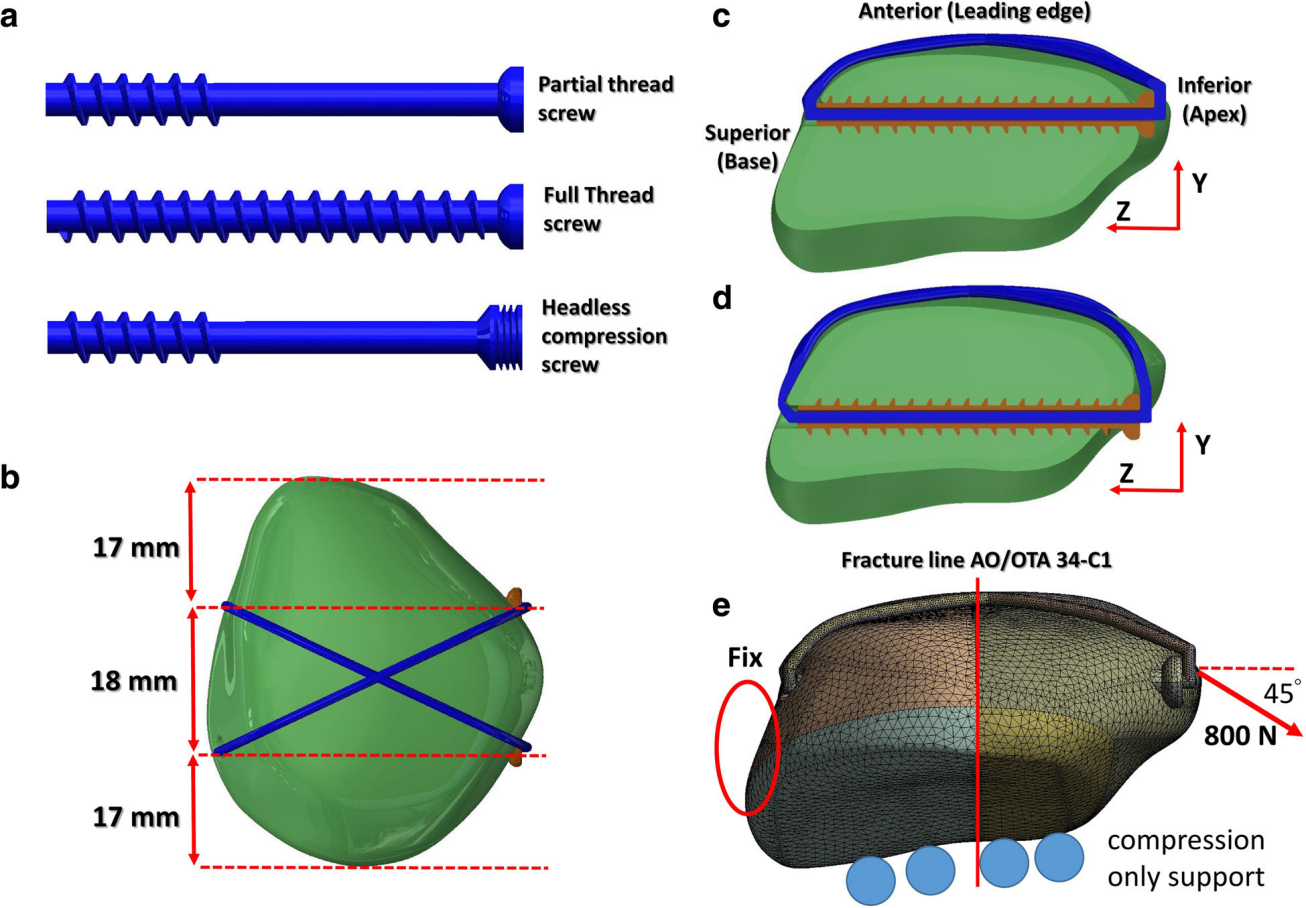
Models used in this study. Screw types (**a**), locations (**b**, **c**, and **d**), and loading directions (**e**)

First, an intact patellar model was created on the basis of computed tomography images obtained from the Visible Human Project of the National Institutes of Health (Bethesda, Maryland, USA). These images were taken at 1-mm intervals. The bony contours in each image were retrieved by thresholding gray values by using Avizo (version 6; VSG SAS, Bordeaux, France). A 3D bony model was rendered using the retrieved bone contours. Subsequently, the model was imported into the CAD software SolidWorks 2014 (Dassault Systemes SolidWorks Corp., Waltham, MA, USA) for fracture creation and screw and wire implantation. In addition, based on the image, a 1-mm sheet was sectioned from the tail edge of the patella to serve as cartilage. A virtual transverse fracture without any gap was created at the midline of the patella (AO/OTA 34-C1 classification). Subsequently, three types of cannulated screws, namely partial thread, full thread, and headless compression screws, were created based on commercial screws (DePuy Synthes, Pennsylvania, USA) and were then inserted from the apex to the base of the fractured patella through “Boolean Operation” in SolidWorks 2014. Two screws were parallelly placed at the middle third of the patella and at 5 or 10 mm away from the anterior surface of the patella (Fig. 1). The outer diameter and length of all screws were set to 4 and 35 mm, respectively. The thread length of both partial thread and headless compression screws was 12 mm. The pitch was set to 2 mm for full- and partial thread screws and the body of the headless compression screw, and the pitch was set to 1 mm for the head of the headless compression screw. To construct a tension band, an anterior wire was bent into a figure of eight on the anterior surface of the patella. The outer diameter of the wire was set to 1.25 mm (DePuy Synthes).

### FE modeling

The solid model was then imported into ANSYS Workbench (version 17; Swanson Analysis, Houston, Pennsylvania, USA) to simulate patella loading during knee flexion/extension. High-order quadratic tetrahedral elements were used to mesh all parts, including the bones, screws, and wire. The mesh density of the screws and wire was locally refined by reducing the length of the element edge by using the command “sizing” in Workbench. The lengths of the global element edge and the refined element edge (the wire and screw) were set to 0.5 and 0.25 mm, respectively. The contact behaviors between the bone and screw, screw and wire, wire and bone, and bone and bone (fracture site) were all set to frictional surface to surface contact (contact 174 and target 170 in ANSYS Workbench), and the frictional coefficients of metal-to-metal, metal-to-bone, and bone-to-bone were set to 0.2, 0.3, and 0.45, respectively [24]. To simulate the responses of the fractured patella fixed without the anterior wire, anterior wire insertion was excluded in the FE model by using the command “Suppress Body” in Workbench, but the other components remained the same.

Material properties of the bone were defined from literature. The elastic moduli of cortical bone, trabecular bone, and cartilage were set to 1000, 207, and 50 MPa, respectively. Their Poisson ratios were set to 0.3, 0.3, and 0.45, respectively [25, 26]. The elastic moduli of bone and cartilage were simplified as linear elastic, isotropic, and homogeneous. Metallic implants, including the screw and wire, were composed of stainless; under plastic material properties, the bilinear hardening effect was selected in the engineering data bank of Workbench. Hence, in the linear elastic stage, the elastic modulus and Poisson ratio were set to 210 GPa and 0.3, respectively; In the plastic stage, the yield strength and tangent modulus were set to 250 MPa and 1450 MPa, respectively.

A tension force was applied to the apex of the patella to simulate the worst case condition of the patella during knee flexion/extension. Loading with two magnitudes was applied because the fractured patella fixed without the anterior wire would not be able to take the same load as the fractured patella fixed with the anterior wire. Therefore, 800-N loading force was applied to the fractured patella fixed with the anterior wire, whereas half loading (400 N) was applied to the fractured patella fixed without the wire [27–29]. The 800-N force was applied in two steps of equal loading: A 400-N force was gradually applied to the fractured patella in the first step, and additional 400 N was applied in the second step. The results of the patella under 400-N force fixed with and without the anterior wire were compared to clarify the effects of the anterior wire. Furthermore, two loading directions, parallel and 45° to the long axis of the patella, were used to simulate patellar loading during knee flexion at different angles.

### Validation and convergence

To validate the present patellar FE model, the results of the fractured patella fixed with a partial thread cannulated screw without the anterior wire were compared with the results of Dargel’s experimental test [28]. In addition, the results with the anterior wire were compared with Bryant’s study [29]. Although the exact screw proximity was not declared in the studies, the results of linear stiffness and a load causing 2-mm deformation were consistent between the present model and the experiment (Dargel’s and Bryant’s), particularly for the patellar fracture fixed with 10-mm screw placement (Table 1).

**Table 1:** Comparison of the stiffness and load with 2 mm deformation in the experiment and FE calculations

Without anterior wire			
	Dargel	Present FE model with 10-mm screw placement	Present FE model with 5-mm screw placement
Loading direction parallel			
Linear stiffness (N/mm)	240.67 ± 28.44	275	879
Load with 2 mm deformation (N)	549.64 ± 56.18	549	1795^a^
Loading direction 45°			
Linear stiffness (N/mm)	147 ± 50.96	129	372
Load with 2 mm deformation (N)	351.35 ± 119.88	280	744^a^
With anterior wire			
	Bryant	Present FE model with 10-mm screw placement	Present FE model with 5-mm screw placement
Loading direction 45°			
Linear stiffness (N/mm)	248.2 ± 30.9	365.3	406.1

^a^means the value obtained from linear extrapolation

### Incidence

In a previous mechanical test of a fractured patella fixed with a metallic implant, the relationship between gap formation and the applied load was analyzed using an optical tracker [30], however, the detailed information including the contact pressure and area were missing. Therefore, in this study not only the maximum displacement of the fractured patellar fragment, maximum gap formed at the fracture site, but also the maximum contact area and pressure at the fracture site after static balance were determined as indices to evaluate the effect of various screw types and locations and anterior wire use and nonuse on the fractured patella. The maximum gap formed and fragment displacement under loading represent the “stability” of the fractured patella with various fixation conditions.

## Results

### Displacement and gap formation

**Fig. 2:**
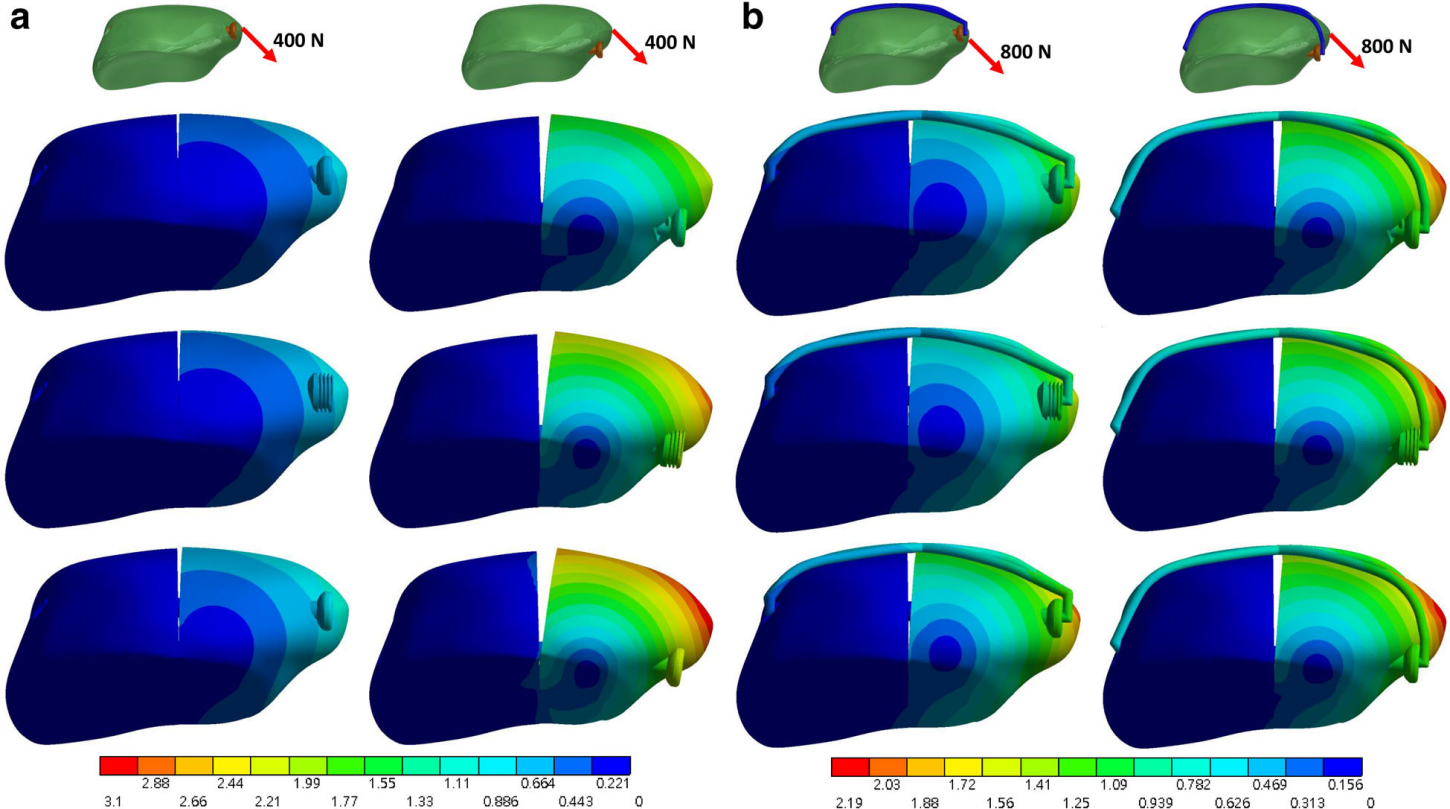
Total deformation (mm) of the fractured patella under a loading force in a direction 45° to the long axis of patella without (**a**) and with (**b**) the wire. The top, middle and bottom row is the full thread, headless compression and partial thread screw, respectively

**Fig. 3:**
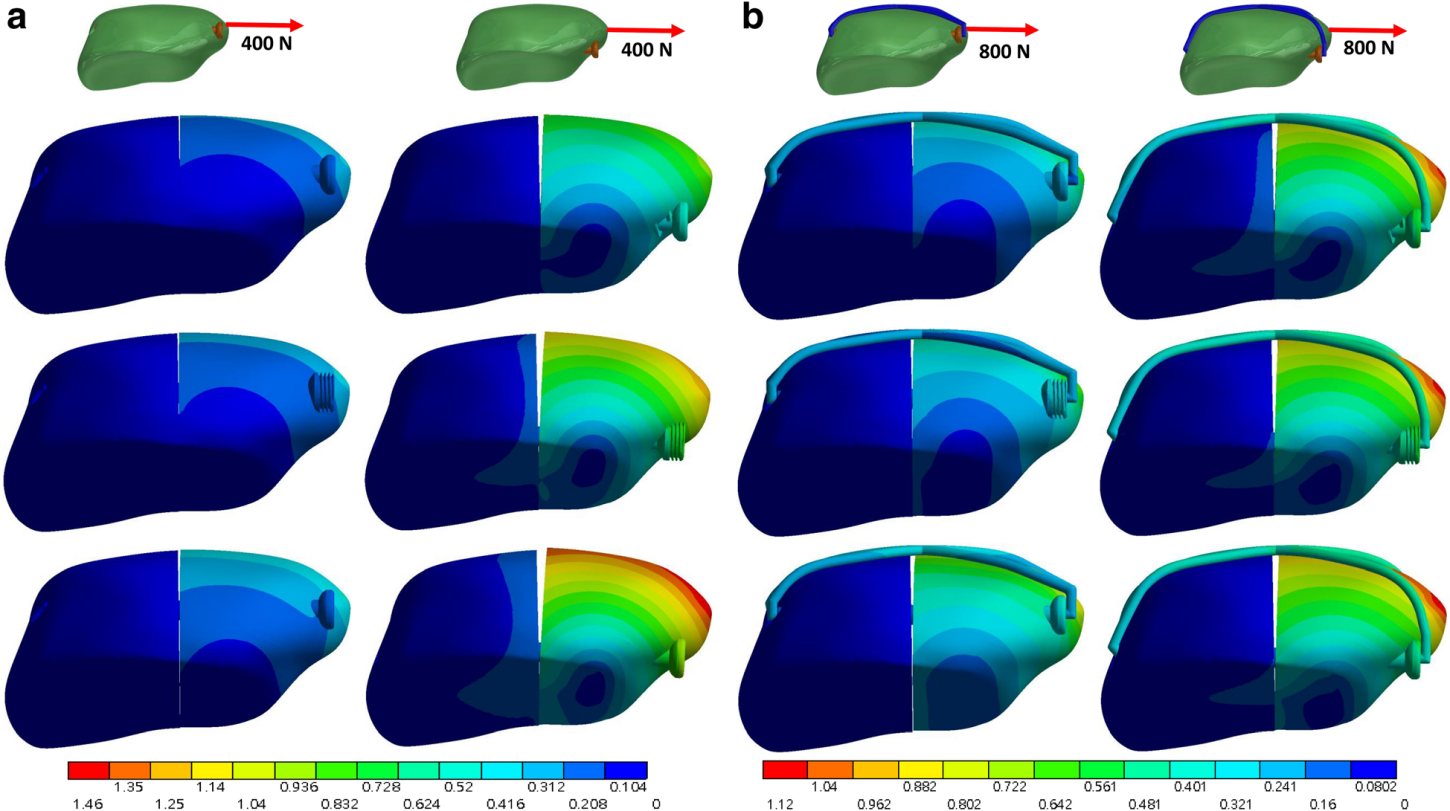
Total deformation (mm) of the fractured patella under a loading force in a direction parallel to the long axis of patella without (**a**) and with (**b**) the wire. The top, middle and bottom row is the full thread, headless compression and partial thread screw, respectively

Among the three screw types investigated in this study, the full thread screws helped to achieve the smallest gap formation and fragment displacement, particular in the setting of a deep screw placement (10-mm) and the absence of the anterior band wiring (Fig. 2 (a) and Fig. 3 (a)). The influences from different screw type on the stability became less evident when the screw proximity was shrunk or the anterior wire was used (Fig. 2 (b), Fig. 3 (b) and Table 2). Additionally, a superficial screw placement (5-mm) also contributed to a smaller gap formation as well as fragment displacement than those obtained by the 10-mm screw placement. Under the load of 400-N in the direction of 45°, the full thread screw with 5-mm placement reduced the gap formation by 86.7% (from 2.71 to 0.36 mm) and 55.6% (from 0.81 to 0.36 mm) compared with the partial thread screw with 10-mm placement, respectively without and with the anterior wire (Table 2).

**Table 2:** Maximum displacement of the fragment, gap opening, contact pressure and contact area of the fractured patella with screw and wire under various loading magnitudes and directions

	Without wire under 400-N			With wire under 400-N			With wire under 800-N		
	Partial thread	Headless compression	Full thread	Partial thread	Headless compression	Full thread	Partial thread	Headless compression	Full thread
Loading direction 45°									
5 mm screw placement Max. fragment displacement (mm)	1.08	0.85	0.79	1	0.84	0.79	1.97	1.54	1.49
Max. gap opening (mm)	0.65	0.43	0.36	0.57	0.4	0.36	1.11	0.7	0.63
Max. contact pressure (MPa)	3.24	2.79	2.54	2.63	2.45	2.32	5.6	5	4.9
Contact area (mm^2^)	192	259	307	205	273	320	180	259	308
10 mm screw placement Max. fragment displacement (mm)	3.1	2.79	2.06	1.24	1.23	1.2	2.19	2.16	2.08
Max. gap opening (mm)	2.71	2.34	1.59	0.81	0.8	0.76	1.36	1.32	1.23
Max. contact pressure (MPa)	9.18	8.85	7.21	3.6	3.5	3.5	6.7	6.5	6.4
Contact area (mm^2^)	94	112	163	129	142	175	135	144	174
Load direction parallel									
5 mm screw placement Max. fragment displacement (mm)	0.45	0.35	0.33	0.45	0.35	0.33	0.86	0.63	0.59
Max. gap opening (mm)	0.35	0.22	0.19	0.35	0.22	0.19	0.67	0.38	0.32
Max. contact pressure (MPa)	0	0.23	0.19	0	0.19	0.17	0	0.39	0.37
Contact area (mm^2^)	0	92	120	0	103	110	0	86	121
10 mm screw placement Max. fragment displacement (mm)	1.46	1.24	0.88	0.75	0.75	0.71	1.12	1.1	1.07
Max. gap opening (mm)	1.35	1.1	0.73	0.63	0.61	0.56	0.86	0.82	0.77
Max. contact pressure (MPa)	2.81	2.8	2.17	0.86	1.3	1.47	1.21	1.67	2.15
Contact area (mm^2^)	63	91	148	44	81	144	33	67	137

### Contact pressure and area

**Fig. 4:**
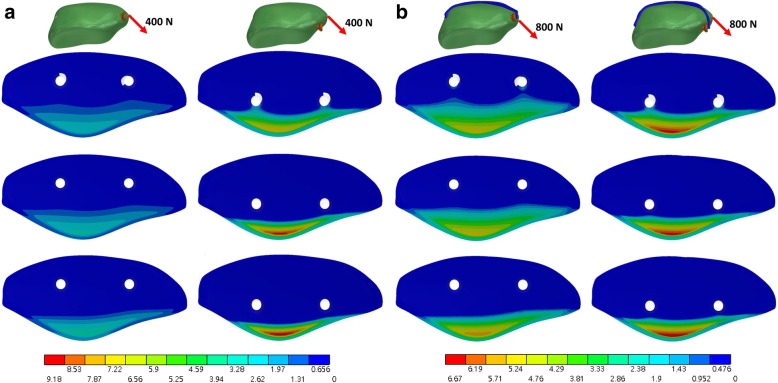
Contact pressure (MPa) of the fracture site under a loading force in a direction 45° to the long axis of the patella without (**a**) and with (**b**) the wire. The top, middle and bottom row is the full thread, headless compression and partial thread screw, respectively

**Fig. 5:**
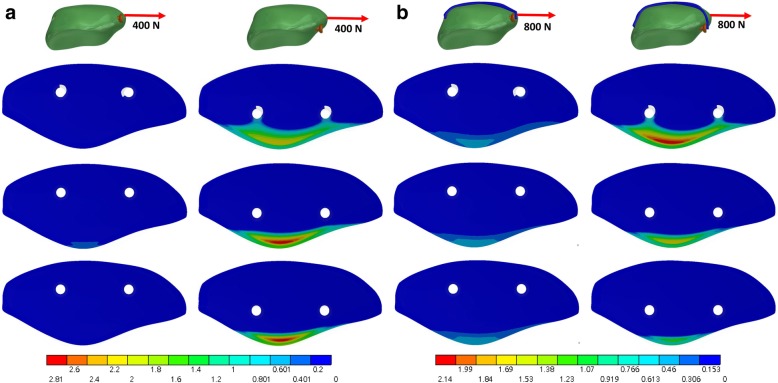
Contact pressure (MPa) of the fracture site under a loading force in a direction parallel to the long axis of the patella without (**a**) and with (**b**) the wire. The top, middle and bottom row is the full thread, headless compression and partial thread screw, respectively

The full thread screw yielded the largest contact area at the fracture site (Table 2) while the partial thread cannulated screw resulted in the smallest contact area. The contact area with 5-mm placement under 800-N force in a direction 45° was 308 and 180 mm^2^ in the full thread screw and partial thread screw, respectively. The full thread screw yield lower contact pressure at the fracture site than the partial thread screw, particular with 10-mm screw placement and without the anterior wire (Fig. 4 (a) and Fig. 5 (a)). Furthermore, the peak pressure decreased with the use of anterior banding wire (Fig. 4 (b) and Fig. 5 (b)). Without the use of anterior wire, the maximum contact pressure obtained by partial thread screws was 9.18 MPa under the load of 400-N force in a direction 45°, while the value decreased to just 3.6 MPa with the existing anterior wire.

## Discussion

This is the first numerical simulation to examine the stability as well as the contact status of the surgical fixation of the transverse patellar fractures using different screws, proximity and anterior wire. Our results indicated the importance of the thread type as well as screw proximity (location) for the mechanical stability and contact area and pressure when the transverse fractured patella was fixed using screws and a wire. Moreover, the anterior wire is critical to maintain the mechanical stability of the fractured patella when screws were placed far away from the anterior surface of the patella; particularly the partial thread screw was chosen. However, the contribution of the anterior wire to stability became less prominent with the decreasing of the screw proximity. These results provide a biomechanical basis for clinicians to make appropriate decision to treat the transverse patellar fractures with MIS or open techniques based on the devices available.

The fractured patella fixed with the screw and wire showed different deformation modes under different loading directions. When the load was applied parallel and close to the screws (5-mm proximity), the whole construction sustained an axial traction force and the fragments separated from each other. In this situation, the full thread screw helps to prevent the fragment from sliding against the screw shaft and led to smaller displacement than the partial thread screw. When the screw was placed far away (10-mm proximity) from the loading site, the loading force generated a torque to the screw, and the fragment demonstrated rotation. When the loading force was applied 45° to the long axis of the patella, both a torque and an axial traction force developed from the loading on each screw location and screw type.

Although more and different screw types were considered in this study, screw proximity affects the stability of the fixation of the fractured patella, including gap formation and fragment displacement. This finding is consistent with the result of our previous study, though only partial thread screw was used in that work: the screw located near the anterior surface of the patella provided higher stability than that located near the posterior surface (the articular surface) [23]; the lower stability provided by the screw located near the posterior surface was due to its placement far away from the rotation center and on the tension side. In the present numerical simulation examining the complex contact status between fragments without sample variance, the difference in the stability of various screw proximities was obvious. By contrast, no significant different gap formation between different screw proximities was observed in a cadaveric biomechanical study by Domby et al. [30]. Thus, we attributed this inconsistency between studies to the variance between samples and the difficultly in determining slight changes under current laboratory setting.

The anterior wire is important for maintaining the reduction of the fractured patella and contact at the fracture site to prevent separation when the screws are placed at 10 mm and sustain a torque (under a loading force in a direction 45° to the long axis of the patella). While the stabilizing effect of the anterior wire on the fractured patella decreased with the screw proximity to the anterior surface of patella. Inserting the anterior wire at the edge of the patella and on the tension side can efficiently reduce the migration of the fractured patellar fragment when it rotates. In addition, such wire insertion increases the moment of inertia of the fractured patella fixed with screws and wire because of its distant placement from the rotation center; subsequently, stability increased. Thus, once surgeons choose the use of deeper screws (10-mm placement) to fix the transverse patellar fracture without anterior wiring, which means the screws are located closer to the rotation center without any resisting device against the bending moment on the patella. Hence, fragment displacement as well as the gap formation becomes obvious, and the contact area decreases under this fixation. When the screw is with 5-mm placement, it shows higher resistance ability to the bending load than the screw with 10-mm placement because of the higher moment of inertia.

In clinical practice, partial thread screws are frequently used to fix the transverse patellar fractures; and the bone fragments slide along the middle shaft of the screw when a force is applied to the patella, resulting in the formation of gap. By contrast, with the use of full thread cannulated screw, the pass of shaft with full threads through the fracture line may reduce the sliding of the bone fragments along the screw; hence, gap formation and fragment displacement obviously decrease. The effect of full threading of screw on the mechanical stability becomes obvious in the setting of deep screw placement and without the anterior wire. Because of no wire sharing the loads, the implanted screws sustain all the loading forces on the fractured patella. Similar to the anterior banding wire, the effect of the screw threading on the stability decreases with the screw proximity to the anterior surface of patella.

The present study has some limitations. First, only the patella bone was modeled, but its articulation with the distal femur condyles was not. Thus, the sliding of the patella on the distal femur was not simulated. Second, in this study, just one fracture type was considered in the present simulation. The results with different fractures types may change. Third, the elastic modulus of bone was simplified as linear elastic, isotropic, and homogeneous, and the morphology of the trabecular bone was not considered. Fourth, both the headless compression and full/partial thread screw was set as a uniform thread shape at the screw shaft; however, this simplification helps to excluding the disturbance from thread shape.

Recently, MIS techniques had been suggested with fewer healing problems than conventional open ones in the literature [19, 31] focused on the beneficial biologic recovery. Without the use of anterior wiring in MIS, a higher stability was obtained by the use of full thread screws at a superficial screw placement (5-mm), and which imply the possible fixation alternative in MIS. However, we acknowledge the results from the current study should be clinically used with caution due to certain inherent limitations, especially the biologic aspect. For example, in the existing osteoporotic bone quality, the superficial screw placement as well as the full thread design, favorable for the mechanical stability in this study, may easily compromise the surgical fixation with implant cut through the fragile knee cap; whereas the deep screw placement along with the anterior wiring seem more secure option in this clinical scenario.

## Conclusion

To surgically treat a transverse patellar fracture, all three factors, the screw type, proximity to the anterior surface and the use of anterior wiring, contribute the mechanical stability of internal fixation. Based on our results, the use of anterior wire along with the full thread screw is preferentially recommended for maintaining the reduction of the transverse patellar fracture. To reduce the surgical trauma during the open anterior wiring, the use of full thread screws at a 5-mm placement is suggested as a MIS fixation alternative based on the similar biomechanical behaviors under loads. At all events, the deep placement of screws without the anterior banding wire is least suggested for the surgical fixation in transverse patellar fractures.

## Abbreviations

FE: Finite Element
MIS: Minimally invasive surgery
